# Sexual satisfaction and function (SatisFunction) survey post-vaginoplasty for transgender and gender diverse individuals: preliminary development and content validity for future clinical use

**DOI:** 10.1093/sexmed/qfaf011

**Published:** 2025-03-08

**Authors:** Amine Sahmoud, Rebekah Russell, Erika Kelley, Elad Fraiman, Carly Goldblatt, Matthew Loria, Kirtishri Mishra, Shubham Gupta, Rachel Pope

**Affiliations:** Department of Obstetrics and Gynecology, University Hospitals Cleveland Medical Center, Cleveland, Ohio 44106, United States; Case Western Reserve University School of Medicine, Cleveland, OH 44106, United States; Department of Obstetrics and Gynecology, University Hospitals Cleveland Medical Center, Cleveland, Ohio 44106, United States; Case Western Reserve University School of Medicine, Cleveland, OH 44106, United States; Department of Urology, University Hospitals Cleveland Medical Center, Cleveland, Ohio 44106, United States; Case Western Reserve University School of Medicine, Cleveland, OH 44106, United States; Case Western Reserve University School of Medicine, Cleveland, OH 44106, United States; Case Western Reserve University School of Medicine, Cleveland, OH 44106, United States; Department of Urology, University Hospitals Cleveland Medical Center, Cleveland, Ohio 44106, United States; Department of Urology, University Hospitals Cleveland Medical Center, Cleveland, Ohio 44106, United States; Department of Urology, University Hospitals Cleveland Medical Center, Cleveland, Ohio 44106, United States

**Keywords:** sexual function and satisfaction (SFS), transgender and gender diverse individuals (TGDI), gender dysphoria, vaginoplasty, community advisory board, anatomy

## Abstract

**Background:**

Transgender and gender diverse individuals (TGDIs) are people whose gender identity is not in line with their sex assigned at birth, but current surveys used for cisgender patients addressing sexual satisfaction and function (SFS) do not fit the needs of this unique population.

**Aim:**

The authors of this project sought to create and validate a new comprehensive survey in North American English that differs from the current options for TGDI post-vaginoplasty.

**Materials and methods:**

Using the current literature on SFS as a foundation, a 26-item survey was created and distributed to 16 TGDI at least 3 months post-vaginoplasty. Feedback and review for content validity took place in the forms of interviews with the 16 TGDI, an expert panel, and the creation of a community advisory board.

**Outcomes:**

Feedback was incorporated to transform the original 26-item questionnaire into a 32-question survey with eight domains, named the SatisFunction Survey Post-Vaginoplasty, which represents the preliminary development and content validity of the survey, with its clinical use not recommended until further validation steps are completed.

**Results:**

Feedback focused on improving the clarity of questions to address sexual vs non-sexual behaviors, providing definitions of terms in the question stems for improved user understanding, including more questions on specific anatomic locations, addressing gender dysphoria as it relates to genital self-image, specifying type of vaginoplasty and only including questions relevant to those with or without a vaginal canal.

**Clinical implications:**

The authors foresee clinical use of the survey for recurrent assessment in the postoperative period as well as post-revision.

**Strength and limitations:**

Community-based research is essential in developing an assessment tool tailored to the unique needs of a specific population. This study presents the findings of preliminary content validation but requires further validation before clinical use, and is limited by a small sample size from a single-site institution.

**Conclusion:**

Future directions involve completing the validation process for the survey with distribution to a larger TGDI population with other validated surveys with a subsequent cohort interview to address construct and divergent validity as well as reliability.

## Introduction

Transgender and gender diverse individuals (TGDI) are people whose gender identity is not in line with their sex and associated gender that was assigned to them at birth.^1^ The term cisgender is used to refer to those whose gender identity is in line with the sex and associated gender that was assigned to them at birth. TGDI and cisgender individuals have vastly different experiences both in life as well as in healthcare.^2^ Discrimination toward TGDI has been well documented both inside and outside of the healthcare setting correlating not only with comparably worse healthcare outcomes but increasing levels of mental health morbidities.^1^^,^^2^ With this in mind, multiple efforts have been made across healthcare settings and specialties to improve medical education for providers and increase healthcare access for TGDI.^3^^,^^4^ Nevertheless, knowledge gaps remain an issue due to the nascent nature of transgender health and surgery, especially with regards to sexual medicine. Further, there is a lack of data on outcomes from medical and surgical interventions, which continue to develop.^5^

The goal of trans-affirming care and the specialty of Transgender Medicine and Surgery is to reduce gender dysphoria and affirm TGDI in who they are sometimes via medically necessary interventions. Gender dysphoria is a feeling of distress experienced by those whose gender assigned at birth is not in line with their gender identity.^6^ With regards to surgical outcomes for TGDI, life satisfaction has been demonstrated to increase postoperatively. In a 2017 literature review, transmasculine individuals who underwent metoidioplasty and phalloplasty reported a 93% and 90% satisfaction rate, respectively.^7^^,^^8^In similar studies, transfeminine individuals reported a 91% satisfaction rate after vaginoplasty.^9^ Nevertheless, there is a paucity of literature regarding satisfaction specific to sexual function for TGDI post-genital reconstruction.

Currently there are several validated surveys that exist for cisgender individuals to assess sexual satisfaction and function. These include the Female Sexual Function Index (FSFI), Sexual Health Inventory for Men, the Female Sexual Distress Scale, the Index of Male Genital Self Image, and the Female Genital Self-Image Scale as examples.^10-14^ Given the clear differences between cisgender anatomy and transgender anatomy post-genital reconstructive surgery, these surveys do not capture several aspects of function and experience for TGDI. For example, satisfaction with vaginal caliber and depth and with cosmetic appearance of the vulva is not addressed in any of the measures available to assess sexual function and satisfaction in women.^15^ To the authors knowledge, the only survey created to assess sexual function and satisfaction for TGDI is the Operated Male to FSFI (oMtFSFI) for transgender women (Supplemental Appendix A).^16^ This survey has been validated only in Italian and comprises seven domains including genital self-image, desire, arousal, lubrication, orgasm, satisfaction, and pain. With this survey in mind, the authors sought to develop a new comprehensive survey in North American English that differs from the oMtFSFI by assessing function of specific anatomic locations for TGDI post-vaginoplasty and is created with input from people of the transgender and gender diverse experience. A completely new survey was not fully constructed for this study due to the strong foundation of the oMtFSFI in assessing sexual function and satisfaction in this population. Nevertheless, the field of transgender medicine and surgery is constantly evolving, and while the oMtFSFI is valuable, it may not fully capture the unique aspects of post-surgical sexual function and satisfaction for transgender individuals. Ongoing reassessment is needed to ensure it remains relevant and reflects current standards and experiences.

The validation process for the survey involves multiple phases, with phases 1–4, which are discussed in this paper, focused on measure development and content validity. These phases include extensive testing groups and complex procedures that require the detailed explanation that is included in this text, but they do not yet constitute full psychometric validation. Further phases, including distribution to larger cohorts and cognitive interviewing with psychometric evaluation, will be conducted in subsequent studies. As such, this paper presents only the preliminary development and content validity of the survey and does not recommend its use for clinical or community application until additional validation steps are completed.

## Materials and methods

**Phase 1.** At a single site institution, University Hospitals Cleveland Medical Center, a panel of experts in Transgender Medicine and Surgery was developed to longitudinally assess the survey throughout its creation and validation with most feedback provided during phase 3. This panel included five experts in psychology, urology, gynecology, and sexual medicine who all had extensive experience in Transgender Medicine and Surgery and were all part of the University Hospital Cleveland Medical Center LGBT and GenderCare Provider Network. Those who remained consistently involved in validation analysis throughout all phases included the principal investigator who is a gynecologist, a sexual medicine trained psychologist, and a gynecology resident. The IRB approved of this study at University Hospitals Cleveland Medical Center (STUDY20221324).

**Figure 1:**
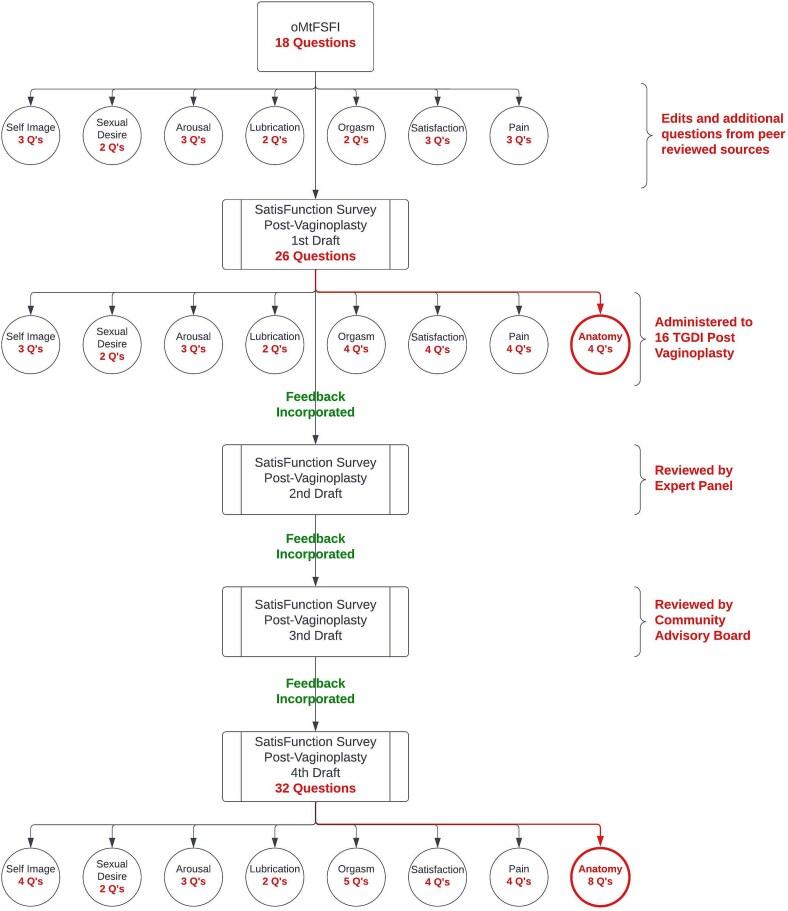
A flowchart depiction of the validation process for study of phases 1–4 of validation as well as a depiction of changes made to number of questions within each domain after each phase.

Using the 18 question oMtFSFI as a foundation, the authors developed the first draft of a 26 question survey, editing the language to fit North American English vernacular and creating additional questions for a more comprehensive overview of sexual function and satisfaction specific to TGDI post-vaginoplasty (Figure 1). These additional questions were created by the expert panel informed by peer reviewed sources and previously validated sexual health surveys intended for cisgender people. This allowed for the assessment of specific aspects of sexual function and satisfaction that were not included in the oMtFSFI. This included topics such as post-surgical scarring, width and depth of the vaginal canal, pain specific to oral sex and masturbation, and sensitivity of specific anatomic locations including the prostate.^17-22^ This phase took place between January and March 2023.

**Phase 2.** The initial survey developed from phase 1 was distributed to *N* = 16 TGDI who had undergone vaginoplasty. These were individuals known to the institution who were receiving regular care. Inclusion criteria included TGDI greater or equal to the age of 18 and must have undergone vaginoplasty at least three months prior to survey administration, to allow for postoperative healing. Participation was advertised via patient visits and phone calls as completely voluntary and could be discontinued at any time. It was also explicitly stated that participation, or lack thereof, would not impact their clinical care.

The survey was administered to this 16-person cohort along with a PHQ-9, originally thought to be used as a possible validation measure (as lower sexual satisfaction and function is strongly correlated with depression symptoms).^23^ The survey and PHQ-9 were distributed electronically via RedCap along with a consent form that was completed prior to taking the surveys. The goal of this step in the validation process was to gather additional stakeholder feedback from the patient population, assess face and content validity, and receive specific feedback on language, comprehension, and any topics that should be included or excluded.

Next, 16 individual interviews were conducted by two research coordinators over phone or zoom, taking written records of the feedback from each participant. The interviews were conducted using cognitive interviewing techniques as a measure of content validity.^24^ Cognitive interviewing bears importance when assessing for participant comprehension of a draft survey such as this, while also verifying that their survey responses correspond with their intention and experience. While cognitive interviewing can manifest in several forms, this study engaged participants during the interview (retrospective to survey completion) to share their thoughts aloud for each question of the survey, demonstrating their understanding of the question, appropriateness of the answer choices, as well as their lived experience that led to their answer choice.^24^ For example, the Anatomy domain includes a question about the sensitivity of the clitoris over the past 4 weeks during sexual activity or intercourse. The participant would or would not confirm understanding by reiterating the question in their own words while also providing information about their postoperative experience with relation to clitoral sensitivity. Interviews also included asking for general feedback about the survey, the participants’ postoperative experience, timing of specific anatomic sensation and orgasm postoperatively, and general postoperative satisfaction or dissatisfaction. This feedback was consolidated and reviewed by the two interviewers for recurrent themes. Feedback was then incorporated via the two interviewers to create a second draft of the survey (Figure 1). Phase 2 interviews were conducted between March and July 2023.

**Phase 3.** The survey developed from phase 2 was then reviewed and edited by a five person expert panel to create a third draft. Members of the expert panel included two reconstructive urologists who perform both transfeminine and transmasculine genital reconstruction, one gynecologist who specializes in sexual medicine for both cisgender women and transgender individuals while also performing vulvoplasty, one gynecology resident with a research focus on transgender health, and one doctorate psychologist with expertise in both sexual medicine and transgender health. Agreement to participate in the study was made at its inception with all experts expressing the dearth of evidence based medicine and inability to assess sexual function in this patient population. All members met over Zoom meetings three times to provide feedback on how to incorporate changes from phases 1 and 2 and create the third draft of the survey (Figure 1). These meetings occurred throughout 2023 and early 2024.

**Phase 4.** A community advisory board (CAB) was created, selecting three transgender women from the greater Cleveland area with diverse personal and surgical backgrounds (Table 2). The authors stress the importance of community based research in allowing the patient population to improve the future of the healthcare they will receive. The goal of the CAB was to provide a final thorough assessment of the survey prior to moving forward with further validation processes. One session was completed with the CAB in May 2024 to thoroughly review the third draft of the survey. Feedback obtained from this session was used to create the fourth draft, a 32 question survey, named the SatisFunction Survey Post-Vaginoplasty (Supplemental Appendix B). This fourth draft marked the end of the content validity portion of this study, but is not the final draft of this survey as further psychometric validation is still required. Clinical use of this fourth draft (Supplemental Appendix B) is not recommended for clinical use. Feedback obtained during this phase was incorporated via a fourth zoom meeting with the expert panel for thorough review and collaboration. The CAB will be reengaged in future validation of the survey when more data is obtained using the fourth draft of the survey (Figure 1).

## Results

As previously mentioned, four overarching phases of the preliminary and content validation process for this study were conducted, incorporating feedback and changes after each step. These included creation of the initial survey as well as feedback from a 16-person cohort of TGDI, an expert panel, and a CAB (Supplemental Appendix D). The first draft of this survey included 26 questions distributed into eight domains (Table 1), seven of which were the same as the oMtFSFI and the eighth being Anatomy for the assessment of the clitoris, prostate, labia minora and labia majora (Supplemental Appendix B).

**Table 1:** Domains of survey, all of which were included in the oMTFSFI, except the domain of anatomy.

Domains
Self-image
Sexual desire
Arousal
Lubrication
Orgasm
Satisfaction
Pain
Anatomy

**Phase 2.** After completion of the survey, the 16 person cohort of TGDI provided individual feedback of the 26 question first draft of the measure during one on one interviews informed by cognitive interviewing methods. Themes derived from the interviews included the recommendation to specify the type of vaginoplasty at the survey start and cater the survey questions to the presence of a vaginal canal or lack thereof (ie, vulvoplasty, zero depth vaginoplasty, etc.) via inclusion or omission of questions. It was also advised to remove the PHQ-9 as a validity measure as it brought up unnecessary mental health issues that did not pertain to their sexual life or gender dysphoria. The inclusion of a question regarding gender dysphoria relative to genital self-image was discussed frequently during interviews with a common sentiment being that there was improvement in gender dysphoria specific to genital self-image post-operatively. More questions regarding sensitivity of anatomic locations were also requested to further specify level of sensation. Moreover, the directions for the survey initially referred to sexual activity as sexual actions or stimulations experienced alone or with a partner (ie, masturbation, sex toys, oral sex, penetrative sex). Participants stated that further defining sexual vs non sexual activity would clarify the mindset required for the survey. Similarly, each question in the original 26 question survey placed a time frame of 4 weeks which was found to be limiting for participants. Finally, a comment section at the end of the survey was recommended by the TGDI cohort for any additional concerns not addressed in the survey. All recommendations aforementioned were incorporated to develop the second draft of the measure, which included 28 items (Supplemental Appendix D).

**Phase 3.** The expert panel reviewed the second draft of the survey to aid with the incorporation of feedback from the 16 person cohort, phase 2. Language used throughout the survey was reassessed for comprehension and inclusivity of a wide range of sexual experiences, positive and negative. Thus, definitions of terms such as gender dysphoria, desire, and arousal were included in the question stem. Additional questions regarding oral and anal sex were also added to multiple domains for a more comprehensive overview of orgasm and pain, for example. Further, the quality and intensity of sensations such as pain and sensitivity of specific anatomic locations were included with additional questions, resulting in a total of 32 items for the third draft of the survey.

**Phase 4.** The CAB concluded this scale development process and further edited language throughout the survey to ensure laypeople comprehension. This was done via a Zoom meeting including members of the research team and the three CAB participants (Table 2). The expert panel reconvened in this phase for discussion and incorporation of the CAB feedback. Via this feedback, the 32 question survey was prefaced with two additional questions (Supplemental Appendix C). The first asked if participants have been sexually active in the past four weeks. Although participants explained that the time frame of four weeks written in each question stem throughout the survey was distracting and limiting, four weeks is still well studied and validated for recall of healthcare issues.^25^ Thus, the authors chose to include it in the survey’s assessment via different formatting. The second question added to preface the survey asked what type of vaginoplasty was performed including “Zero Depth/Vulvoplasty, Shallow Depth, Penile Inversion, Peritoneal Pull Through, Intestinal/Colon, Other, and I’m Not Sure” options. Via RedCap branching logic, questions dealing with a vaginal canal in any capacity were omitted if “Zero Depth/Vulvoplasty” or “Shallow Depth” options were selected. This resulted in a total of 32 items for the fourth draft version of the survey with two additional prefacing questions.

**Table 2:** Demographics of members of the community advisory board. Age ranges done for anonymity.

Age	Race	Sex assigned at birth	Gender identity	Relationship status	Type of vaginoplasty
60–65	White	Male	Trans woman	Single	Peritoneal pull through
30–35	Black	Male	Trans woman	Single	Penile inversion
60–65	White	Male	Trans woman	Married	Shallow depth

**Domains of the fourth draft 32 item survey.** The following is a description of the constructs assessed in the final version of this scale summarizing the four phases of feedback and incorporation of that feedback (Supplemental Appendix D).

**Domain 1: genital self-image.** The first domain is genital self-image and includes questions regarding comfort with appearance when alone and with a partner, similar to oMtFSFI. In contrast to gender dysphoria, which is a common experience for TGDI, many of the 16 participants from phase 2 described a feeling of joy and satisfaction they experienced while visualizing their new genitalia post-operatively, a feeling commonly known as gender euphoria. Notably, this experience was reported even in people who had postoperative complications. In order to more holistically assess the range of experiences with genital self-image, questions regarding comfort with post-surgical scarring, gender euphoria, and gender dysphoria were also added to this section after phase 2 was completed.^6^^,^^19^ A definition of gender dysphoria (ie, discomfort or stress related to gender) was also included in the question stem to facilitate comprehension after discussion with the expert panel during phase 3.

**Domain 2: desire.** Definitions for sexual desire (ie, the feeling of wanting a sexual experience, feeling receptive to a partner’s sexual initiation, or fantasizing about having sex) were added into the question stems as part of the feedback from the expert panel during phase 3.

In comparison with the oMtFSFI, questions within the desire section were reworded during phase 3 to assess frequency and satisfaction with level of desire.

**Domain 3: arousal.** Definitions for sexual arousal (ie, the mental/physical feelings of sexual excitement, warmth, or tingling in the genitals, muscle contractions) were again mentioned in the question stem for improved comprehension as part of phase 3 feedback. Questions about arousal were modified to assess satisfaction with frequency of arousal rather than assessing frequency alone again during phase 3. Questions about arousal also include both intensity and satisfaction with that intensity. The authors believe that assessing satisfaction specific to frequency and intensity within these domains provides personal information on quality of life rather than assessing frequency and intensity as an independent factor.^26^

**Domain 4: lubrication.** When assessing lubrication, the authors differentiate between exogenous and endogenous lubrication. During interviews with the 16 person cohort during phase 2, multiple participants disclosed experiencing unwanted secretions or discharge outside of sexual intercourse. Thus, rather than assessing lubrication during sexual activity alone, secretions both during and outside of sexual activity are assessed as a measure of neovaginal function.

**Domain 5: orgasm.** Orgasms are subjectively assessed and may be felt in multiple locations externally and internally including the clitoris, vagina, and anus.^22^ In interviewing the 16 person cohort during phase 2, these anatomic locations were discussed as areas that lead to orgasm with stimulation. After discussion with the expert panel during phase 3, questions about the ability to orgasm and quality of those orgasms were included in the survey. Additional questions assessing ability to achieve orgasm with clitoral, vaginal, or anal stimulation were also included as part of the feedback from phase 3.^22^ Anatomic sensitivity and function postoperatively was an aspect the authors wanted to capture within this survey as it is not currently represented in its counterparts.^10-14^^,^^22^

**Domain 6: satisfaction.** The sixth domain is satisfaction and assesses general satisfaction with sex life. Questions included satisfaction with sexual activity generally as well as vaginal penetration. Moreover, the creation of the survey during phase 1 implemented the delineation between depth and width of the vagina rather than asking about size alone. This added specificity could possibly guide future medical or surgical management.

**Domain 7: pain.** Sexual pain is the seventh domain. Again, specifying the anatomic location, especially with regards to pain, can prove to be beneficial in targeting therapies to improve quality of life. The oMtFSFI assesses pain with regards to vaginal penetration alone. While this is an important part of the assessment, it is not inclusive of those without a vaginal canal or those who experience pain in different locations of the vulva or canal with other sexual activities. In the 16 person cohort of phase 2, several participants disclosed that they experience pain with clitoral stimulation at times. With this in mind, questions regarding pain and intensity of that pain were included for both vaginal penetration and clitoral stimulation, bearing in mind that patients can answer the questions based on experiences with masturbation or sexual activity with others.

**Domain 8: anatomy.** The final domain focuses on anatomy. The authors stress the importance of targeted anatomy questions in surveys such as these to more specifically assess function. Creation of the survey during phase 1 included asking about sensitivity of different anatomic structures. Phase 3 then included defining “sensitivity” as awareness of light touch within the question stem. Due to the subjectivity of this question, a follow up question of satisfaction with that sensitivity was also included for each anatomic structure as a result of phase 3 deliberation. These structures included the clitoris, labia majora, labia minora, and prostate. “Sensitivity” for the prostate was defined as awareness of touch or pressure given its unique anatomic location for those with vaginoplasty. With these questions, a more holistic and specific assessment of function can be completed postoperatively.

## Discussion

An initial 26 question survey was modified to create the SatisFunction Survey Post-Vaginoplasty, a 32 question comprehensive survey to assess sexual function and satisfaction in TGDI post-vaginoplasty. This was done via a validation protocol of 4 iterative phases, including feedback from a 16 person cohort of TGDI individuals, an expert panel, and a CAB. These phases of the study provide preliminary and content validation for a measure to assess sexual function post-GAS, a critical domain of health and wellbeing. Valuable information was obtained through the assessments and analyses of the survey by the different groups of each phase. As previously mentioned, the initial 26 question survey was created utilizing the oMtFSFI as a base. The seven domains of the oMtFSFI were again included in this study’s survey with the addition of an Anatomy Domain (Table 1). Each domain of the survey was carefully analyzed throughout each phase of the study for optimal comprehension and holistic coverage of each aspect of sexual function and health (Supplemental Appendix B, Supplemental Appendix C, Supplemental Appendix D, Table 1).

Strengths of this study include community participation from the transgender and gender diverse population of Cleveland, which is integral to the development of the survey via incorporation of key stakeholder feedback. The addition of a domain with questions targeting specific anatomy also strengthens the assessment of sexual function and satisfaction for this population. Limitations of this study include the inclusion of a sample from a specific geographic region, and results may not be generalizable to the larger US population, a focus for future studies. In addition, this study was limited in its focus on face, content, and criterion validity. Future psychometric assessment of validity and reliability is warranted prior to its use clinically. While this part of the study created a fourth draft of the survey utilizing community input, reliability has not been assessed for this draft (Supplemental Appendix C), nor has it been contrasted to other validated surveys, albeit there is no current gold standard validated survey for this patient population in relation to sexual satisfaction and function post-vaginoplasty. Thus, the authors emphasize that the fourth draft of the survey is not suitable for clinical use in assessing sexual satisfaction and function of TGDI individuals post-vaginoplasty until comprehensive psychometric evaluations and outcome measurements are conducted and published in forthcoming studies. The authors determined that the extensive content validation conducted during the first four phases, as detailed in this manuscript, necessitated a transparent and comprehensive description. This approach ensures clarity and rigor before addressing the psychometric evaluations and outcome measurement processes in the subsequent validation phases, rather than attempting to consolidate all steps into a single paper.

Future directions based upon this study involve completing the validation process for the survey (Figure 1). Some such remaining steps of the validation protocol will include cognitive interviewing of a larger cohort of TGDI, to further refine the items, ensure items are comprehensible, and identify any remaining sources of error. Distribution of the final SatisFunction Survey Post-Vaginoplasty to a larger sample of TGDI will be conducted to examine the factor structure of the survey and to examine divergent validity of the survey via assessment of the correlation of survey scores with validated measures of sexual distress and gender dysphoria.

The authors foresee wide distribution and use of the survey by national and international institutions treating TGDI post-vaginoplasty. Although the survey has clear use in future research studies, the authors also envision its clinical use postoperatively at multiple time points (ie, 3 months, 6 months, 1 year) as well as post-revision to continuously assess for areas of improvement as well as successes or failures of medical and surgical interventions. It is important to include as comprehensive and as community-informed a measure as possible for future clinical and research use.

## Supplementary Material

Supplemental_Appendix_qfaf011
