# Supplementary material for: Sexual satisfaction and function (SatisFunction) survey post-vaginoplasty for transgender and gender diverse individuals: preliminary development and content validity for future clinical use
Source: Sex Med. 2025 Mar 8;13(1):qfaf011. doi: 10.1093/sexmed/qfaf011 (PMC11890106; doi:10.1093/sexmed/qfaf011)
Supplement: Supplemental_Appendix_qfaf011 [file supplemental_appendix_qfaf011.zip › Supplemental_Appendix_qfaf011/Appendix D Question Revisions.docx]

| **Domain**  *Appendix D: Survey Question Revisions by Domain Pre and Post Feedback* | **Pre Feedback** | **Post Feedback*** | **Reasons for changes** |
| --- | --- | --- | --- |
|  | - | Added the question “What type of vaginoplasty did you receive?” | The type of procedure makes questions more or less applicable with omission of questions referring to a vaginal canal if vulvoplasty or shallow depth is selected **(Phase 4)** |
|  | - | Added a singular question “have you been sexually active in the past 4 weeks?” | Participants felt that 4 weeks was a limited time frame so it was removed from each question but included as its own due to previous validation for healthcare recall **(Phase 4)** |
| **Genital Self Image** | 1. Over the past 4 weeks, how often did you feel comfortable with the physical appearance of your external genitalia? | 1. How comfortable are you with the physical appearance of your external genitalia? | Reassessed for comprehension and inclusivity of a wide range of sexual experiences, positive and negative **(Phase 2)** |
| **Genital Self Image** | 2. Over the past 4 weeks, how comfortable did you feel with your partner(s) seeing your external genitalia? | 2. How comfortable do you or would you feel with your partner(s) seeing your external genitalia? | Reassessed for comprehension and inclusivity of a wide range of sexual experiences, positive and negative. **(Phase 2)** |
| **Genital Self Image** | 3. Over the past 4 weeks, how often did scarring interfere with how comfortable you were with the physical appearance of your external genitalia? | 3. How often does scarring interfere with how comfortable you are with the physical appearance of your external genitalia? | Reassessed for comprehension and inclusivity of a wide range of sexual experiences, positive and negative. **(Phase 2)** |
| **Genital Self Image** | - | 4. Compared to before your procedure, rate your gender dysphoria in relation to your genitalia.  (Gender dysphoria means discomfort or stress related to gender) | Added the question due to participant feedback focused on delineating how gender dysphoria changed before and after the procedure **(Phase 3)** |
| **Desire** | 4. Over the past 4 weeks, how often did you feel sexual desire? | 5. How often do you feel sexual desire?  (the feeling of wanting a sexual experience, feeling receptive to a partner’s sexual initiation, or fantasizing about having sex) | Reassessed for comprehension and inclusivity of a wide range of sexual experiences, positive and negative. Definitions of terms were also incorporated for improved understanding. **(Phase 3)** |
| **Desire** | 5. Over the past 4 weeks, how often did you act on your sexual desire (i.e. masturbation, sexual encounters, other sexual events)? | 6. How satisfied are you with your level of sexual desire? | Feedback acknowledged that individuals may feel desire without ability to or wish to act on it. It was recommended to focus on the level of desire separate from acting on it. **(Phase 3)** |
| **Arousal** | 6. Over the past 4 weeks, during sexual activity or intercourse, how often did you feel sexually aroused? | 7. How satisfied are you with the frequency of your sexual arousal?  (the mental/physical feelings of sexual excitement, warmth or tingling in the genitals, muscle contractions) | Feedback acknowledged that individuals may experience sexual arousal outside of sexual activity that may or may not be accompanied by sexual activity. Definitions of terms were also incorporated for improved understanding. **(Phase 3)** |
| **Arousal** | 7. Over the past 4 weeks, during sexual activity or intercourse, how would you rate your level of sexual arousal? | 8. How would you rate the intensity of your sexual arousal? | Feedback acknowledged that individuals may experience sexual arousal outside of sexual activity that may or may not be accompanied by sexual activity. **(Phase 3)** |
| **Arousal** | 8. Over the past 4 weeks, during sexual activity or intercourse, how satisfied were you with your level of arousal? | 9. How satisfied are you with the intensity of your sexual arousal? | Feedback acknowledged that individuals may experience sexual arousal outside of sexual activity that may or may not be accompanied by sexual activity. **(Phase 3)** |
| **Lubrication** | 9. Over the past 4 weeks, how often did you use lubricants during sexual activity or intercourse? | 10. How often do you feel secretions during sexual activity or intercourse without using lubricants?  (engaging in sexual activity alone or with a partner) | Participants stated that further defining sexual vs non sexual activity would clarify the mindset required for the survey. **(Phase 2)** |
| **Lubrication** | 10. Over the past 4 weeks, how often did you feel lubricated (wet) during sexual activity or intercourse without using lubricants? | 11. How often do you feel unwanted secretions outside of sexual activity or intercourse? | Added due to participants expressing lubrication during sexual and non-sexual activities. **(Phase 2)** |
| **Orgasm** | 11. Over the past 4 weeks, how often have you been able to have an orgasm when you wanted to? | 12. How often have you been able to have an orgasm when you wanted to? | Reassessed for comprehension and inclusivity of a wide range of sexual experiences, positive and negative. **(Phase 2)** |
| **Orgasm** | 12. Over the past 4 weeks, how satisfied did you feel with the quality of your orgasm during sexual stimulation and/or intercourse? | 13. How satisfied do you feel with the quality of your orgasms during sexual stimulation and/or intercourse? | Reassessed for comprehension and inclusivity of a wide range of sexual experiences, positive and negative. **(Phase 2)** |
| **Orgasm** | 13. Over the past 4 weeks, have you been able to achieve an orgasm with vaginal penetration? | 14. How often have you been able to achieve an orgasm with vaginal penetration? | Reassessed to further specify level of sensation and sensitivity of specific anatomic locations. **(Phase 3)** |
| **Orgasm** | 14. Over the past 4 weeks, have you been able to achieve an orgasm with clitoral stimulation? | 15. How often have you been able to achieve an orgasm with clitoral stimulation? | Reassessed to further specify level of sensation and sensitivity of specific anatomic locations. **(Phase 3)** |
| **Orgasm** | - | 16. How often have you been able to achieve an orgasm with anal penetration? | Added for a more comprehensive overview of orgasm and pain. **(Phase 3)** |
| **Satisfaction** | 15. Over the past 4 weeks, how satisfied were you with the level of your sexual activity? | 17. How satisfied are you with the amount of sexual activity you have? | Reassessed for comprehension and inclusivity of a wide range of sexual experiences, positive and negative. **(Phase 2)** |
| **Satisfaction** | 16. Over the past 4 weeks, how satisfied were you with vaginal penetration? | 18. How satisfied are you with the feeling of vaginal penetration? | Reassessed to further specify level of sensation and sensitivity of specific anatomic locations. **(Phase 2)** |
| **Satisfaction** | 17. Over the past 4 weeks, how satisfied were you with the width of your vagina (or the ability of your vagina to accommodate what you are putting in it)? | 19. How satisfied are you with the width of your vagina?  (the ability of your vagina to accommodate what you are putting in it) | Definition added to increase understanding and the mindset needed to answer. **(Phase 3)** |
| **Satisfaction** | 18. Over the past 4 weeks, how satisfied were you with the depth of your vagina? | 20. How satisfied are you with the depth of your vagina? | Reassessed to further specify level of sensation and sensitivity of specific anatomic locations. **(Phase 2)** |
| **Pain** | 19. Over the past 4 weeks, how often did you experience pain during vaginal penetration? | 21. How often do you experience pain during vaginal penetration? | Reassessed to address the quality and intensity of sensations such as pain and sensitivity of specific anatomic locations. **(Phase 2)** |
| **Pain** | 20. Over the past 4 weeks, how would you rate your level of pain during vaginal penetration? | 22. How would you rate the intensity of pain you experience during vaginal penetration? | Reassessed to address the quality and intensity of sensations such as pain and sensitivity of specific anatomic locations. **(Phase 2)** |
| **Pain** | 21. Over the past 4 weeks, how often did you experience pain while receiving oral sex? | 23. How often do you experience pain with clitoral stimulation? | Reassessed to address the quality and intensity of sensations such as pain and sensitivity of specific anatomic locations. **(Phase 2)** |
| **Pain** | 22. Over the past 4 weeks, how often did you experience pain with masturbation? | 24. How would you rate the intensity of pain you experience with clitoral stimulation? | Reassessed to address the quality and intensity of sensations such as pain and sensitivity of specific anatomic locations. **(Phase 2)** |
| **Anatomy** | 23. Over the past 4 weeks, during sexual activity or intercourse, how would you rate the sensitivity of your clitoris? | 25. How would you rate the sensitivity of your clitoris?  (sensitivity describes awareness of light touch) | Added to further specify level of sensation. **(Phases 1 and 3)** |
| **Anatomy** | - | 26. How satisfied are you with the sensitivity of your clitoris?  (sensitivity describes awareness of light touch) | Added to increase clarity and identification of specific anatomic locations. Definition added to increase understanding and the mindset needed to answer. **(Phases 1 and 3)** |
| **Anatomy** | 24. Over the past 4 weeks, during sexual activity or intercourse, how would you rate the sensitivity of your labia majora (outer lips)? | 27. How would you rate the sensitivity of your labia majora (outer lips of the vagina)?  (sensitivity describes awareness of light touch) | Reassessed to further specify level of sensation and sensitivity of specific anatomic locations. Definition added to increase understanding and the mindset needed to answer. **(Phases 1 and 3)** |
| **Anatomy** | - | 28. How satisfied are you with the sensitivity of your labia majora (outer lips of vagina)?  (sensitivity describes awareness of light touch) | Added to increase clarity and identification of specific anatomic locations. Definition added to increase understanding and the mindset needed to answer. **(Phases 1 and 3)** |
| **Anatomy** | 25. Over the past 4 weeks, during sexual activity or intercourse, how would you rate the sensitivity of your labia minora (inner lips)? | 29. How would you rate the sensitivity of your labia minora (inner lips of the vagina)?  (sensitivity describes awareness of light touch) | Reassessed to further specify level of sensation and sensitivity of specific anatomic locations. Definition added to increase understanding and the mindset needed to answer. **(Phases 1 and 3)** |
| **Anatomy** | - | 30. How satisfied are you with the sensitivity of your labia minora (inner lips of vagina)?  (sensitivity describes awareness of light touch) | Added to increase clarity and identification of specific anatomic locations. Definition added to increase understanding and the mindset needed to answer. **(Phases 1 and 3)** |
| **Anatomy** | 26. Over the past 4 weeks, during vaginal penetration, how would you rate the sensitivity of your prostate or erectile tissue within your vagina? | 31. During vaginal penetration, how would you rate the sensitivity of your prostate (erectile tissues between bladder and rectum)?  (sensitivity describes awareness of touch/pressure) | Reassessed to further specify level of sensation and sensitivity of specific anatomic locations. Definition added to increase understanding and the mindset needed to answer. **(Phases 1 and 3)** |
| **Anatomy** | - | 32. How satisfied are you with the sensitivity of your prostate (erectile tissue between bladder and rectum)?  (sensitivity describes awareness of touch/pressure) | Added to increase clarity and identification of specific anatomic locations. Definition added to increase understanding and the mindset needed to answer. **(Phases 1 and 3)** |
|  | N/A | Any comments of concerns? Please feel free to share any comments about your sexual journey | Recommended for any additional concerns not addressed in the survey  **(Phase 2)** |
|  | PHQ-9 | N/A | Created mental and emotional distress that was not related to gender dysphoria or sexual satisfaction. **(Phase 2)** |

* Post Feedback includes 16 person cohort, expert panel, and community advisory board

*”-” indicates that it is not present

*Appendix D: This appendix demonstrates the changes and additions made to specific questions within each domain of the survey via the 16 person cohort, expert panel, and community advisory board with explanations for the changes/addition and the respective Phase during which these changes were incorporated*
