# Supplementary material for: Sexual satisfaction and function (SatisFunction) survey post-vaginoplasty for transgender and gender diverse individuals: preliminary development and content validity for future clinical use
Source: Sex Med. 2025 Mar 8;13(1):qfaf011. doi: 10.1093/sexmed/qfaf011 (PMC11890106; doi:10.1093/sexmed/qfaf011)
Supplement: Supplemental_Appendix_qfaf011 [file supplemental_appendix_qfaf011.zip › Supplemental_Appendix_qfaf011/Supplemental Appendix A oMtFSFI translated into English.pdf]

oMtFSI: operated male to female sexual function index

Domain 1: Genital self image.

1. **Over the past 4 weeks, how often did you feel comfortable with your external genitalia?**

1) Almost always or always
2) Most of the time
3) Sometimes
4) Never or almost never

2. **Over the past 4 weeks, how often did you feel satisfied with the aesthetic appearance of your external genitalia?**

1) Almost always or always
2) Most of the time
3) Sometimes
4) Never or almost never

3. **Over the past 4 weeks, how often did you feel comfortable to appear completely naked to your partner?**

1) Almost always or always
2) Most of the time
3) Sometimes
4) Never or almost never

Domain 2: Desire

4. **Over the past 4 weeks, how often did you feel sexual desire?**

1) Almost always or always
2) Most of the time
3) Sometimes
4) Never or almost never

5. **Over the past 4 weeks, how often did you realize your sexual desire?**

1) Almost always or always
2) Most of the time

3) Sometimes
4) Never or almost never

Domain 3: Arousal

6. Over the past 4 weeks, during sexual activity or intercourse, how often did you feel sexually aroused?

1) Almost always or always
2) Most of the time
3) Sometimes
4) Never or almost never

7. Over the past 4 weeks, during sexual activity or intercourse, how would you rate your level of sexual arousal?

1) High
2) Moderate
3) Low
4) Very low or absent

8. Over the past 4 weeks, during sexual activity or intercourse, how satisfied were you with your level of arousal?

1) Very satisfied
2) Moderately satisfied
3) Slightly satisfied
4) Not satisfied at all

Domain 4: Lubrication

9. Over the past 4 weeks, how often did you use lubricants during sexual activity or intercourse?

4) Almost always or always
3) Most of the time
2) Sometimes
1) Never or almost never

10. Over the past 4 weeks, how often did you feel lubricated (wet) during sexual activity or intercourse without using lubricants?

1) Almost always or always
2) Most of the time

3) Sometimes
4) Never or almost never

Domain 5: Orgasm

11. Over the past 4 weeks, how often have you reached the orgasm during sexual stimulation and/or intercourse?

1) Almost always or always
2) Most of the time
3) Sometimes
4) Never or almost never

12. Over the past 4 weeks, how often did you feel satisfied with your orgasm during sexual stimulation and/or intercourse?

1) Almost always or always
2) Most of the time
3) Sometimes
4) Never or almost never

Domain 6: Satisfaction

13. Over the past 4 weeks, how often did you feel satisfied with your sexual activity?

1) Almost always or always
2) Most of the time
3) Sometimes
4) Never or almost never

14. Over the past 4 weeks, how often did you feel satisfied with your vaginal penetrations?

1) Almost always or always
2) Most of the time
3) Sometimes
4) Never or almost never

15. Over the past 4 weeks, how often did you feel satisfied with the size of your neo-vagina?

1) Almost always or always
2) Most of the time

3) Sometimes
4) Never or almost never

Domain 7: Sexual Pain

16. Over the past 4 weeks, how often did you experience pain during vaginal penetration?

1) Almost always or always
2) Most of the time
3) Sometimes
4) Never or almost never

17. Over the past 4 weeks, how would you rate your level of pain during vaginal penetration?

1) Severe
2) Moderate
3) Low
4) Very low or absent

18. Over the past 4 weeks, how often did you have to interrupt a vaginal penetration due to sexual pain?

1) Almost always or always
2) Most of the time
3) Sometimes
4) Never or almost never

© **All rights reserved to oMtFSFI study group** (F. Vedovo, L. Di Blas, C. Perin, N. Pavan , M. Zatta, S. Bucci, G. Morelli, A. Cocci , A. Delle Rose, S. Carossai Grisanti, G. Gentile, F. Colombo, L. Rolle, M. Timpano, P. Verze, L. Spirito, F. Schiralli, C Bettocchi, G. Garaffa, A. Palmieri, V. Mirone,, C. Trombetta). **Operated Male To Female Sexual Function Index (oMtFSFI): a study on the validity of the first questionnaire developed in order to assess the sexual function after male to female gender reassignment surgery** (paper under submission).

Preliminary range values for discriminating normal from progressively critical levels in MtF patients.

	Sexual dissatisfaction	Sexual pain	Genital self-image	Total score
Normal range	10-25	4-6	4-5	18-36
Mild to moderate range	26-33	7-10	6-9	37-49
Borderline critical range	34-35	11	10-12	50-55
Critical range	> 35	> 11	> 12	> 55

© **All rights reserved to oMtFSFI study group** (F. Vedovo, L. Di Blas, C. Perin, N. Pavan , M. Zatta, S. Bucci, G. Morelli, A. Cocci , A. Delle Rose, S. Carossai Grisanti, G. Gentile, F. Colombo, L. Rolle, M. Timpano, P. Verze, L. Spirito, F. Schiralli, C Bettocchi, G. Garaffa, A. Palmieri, V. Mirone,, C. Trombetta). **Operated Male To Female Sexual Function Index (oMtFSFI): a study on the validity of the first questionnaire developed in order to assess the sexual function after male to female gender reassignment surgery** (paper under submission)
