# Supplementary material for: Sexual satisfaction and function (SatisFunction) survey post-vaginoplasty for transgender and gender diverse individuals: preliminary development and content validity for future clinical use
Source: Sex Med. 2025 Mar 8;13(1):qfaf011. doi: 10.1093/sexmed/qfaf011 (PMC11890106; doi:10.1093/sexmed/qfaf011)
Supplement: Supplemental_Appendix_qfaf011 [file supplemental_appendix_qfaf011.zip › Supplemental_Appendix_qfaf011/Supplemental Appendix B 26 Question Survey Pre Feedback.pdf]

Sexual Satisfaction Survey Post Vaginoplasty

Please complete the survey below.

Thank you!

Please fill out all questions below to the best of your ability. You can save and return to the survey using the link in your email if needed. When the survey is completed in its entirety, please click submit at the bottom of the page. We will contact you for the second part of the study, which is interviewing you about these survey questions. Thank you for your time!

Sexual Satisfaction and Function Post Vaginoplasty

This is a survey that will help gauge your sexual satisfaction and function since your vaginoplasty procedure. Each of the following questions will ask you about different aspects of your sexual satisfaction and function and refers to sexual actions or stimulations you have alone or with a partner (e.g. masturbation, sex toys, oral sex, penetrative sex, etc). Please answer the following questions based on how you feel now.

1 Over the past 4 weeks, how often did you feel comfortable with the physical appearance of your external genitalia?

- ◯ Almost always or always
- ◯ Most of the time
- ◯ Sometimes
- ◯ Never or almost never
- ◯ I'm not sure/I don't know

2 Over the past 4 weeks, how comfortable did you feel with your partner(s) seeing your external genitalia?

- ◯ Almost always or always
- ◯ Most of the time
- ◯ Sometimes
- ◯ Never or almost never
- ◯ I'm not sure/I don't know
- ◯ N/A I don't have any partner(s) currently

3 Over the past 4 weeks, how often did scarring interfere with how comfortable you were with the physical appearance of your external genitalia?

- ◯ Almost always or always
- ◯ Most of the time
- ◯ Sometimes
- ◯ Never or almost never
- ◯ I'm not sure/I don't know

4 Over the past 4 weeks, how often did you feel sexual desire?

- ◯ Almost always or always
- ◯ Most of the time
- ◯ Sometimes
- ◯ Never or almost never
- ◯ I'm not sure/I don't know

5 Over the past 4 weeks, how often did you act on your sexual desire (i.e. masturbation, sexual encounters, other sexual events)?

- ◯ Almost always or always
- ◯ Most of the time
- ◯ Sometimes
- ◯ Never or almost never
- ◯ I'm not sure/I don't know

6 Over the past 4 weeks, during sexual activity or intercourse, how often did you feel sexually aroused?

- ◯ Almost always or always
- ◯ Most of the time
- ◯ Sometimes
- ◯ Never or almost never
- ◯ I'm not sure/I don't know

7	Over the past 4 weeks, during sexual activity or intercourse, how would you rate your level of sexual arousal?	◯ High<br>◯ Moderate<br>◯ Low<br>◯ Very low or absent<br>◯ I'm not sure/I don't know
8	Over the past 4 weeks, during sexual activity or intercourse, how satisfied were you with your level of arousal?	◯ Very satisfied<br>◯ Moderately satisfied<br>◯ Slightly satisfied<br>◯ Not satisfied at all<br>◯ I'm not sure/I don't know
9	Over the past 4 weeks, how often did you use lubricants during sexual activity or intercourse?	◯ Almost always or always<br>◯ Most of the time<br>◯ Sometimes<br>◯ Never or almost never<br>◯ I'm not sure/I don't know
10	Over the past 4 weeks, how often did you feel lubricated (wet) during sexual activity or intercourse without using lubricants?	◯ Almost always or always<br>◯ Most of the time<br>◯ Sometimes<br>◯ Never or almost never<br>◯ I'm not sure/I don't know<br>◯ N/A, I always need to use lubrication
11	Over the past 4 weeks, how often have you been able to have an orgasm when you wanted to?	◯ Almost always or always<br>◯ Most of the time<br>◯ Sometimes<br>◯ Never or almost never<br>◯ I'm not sure/I don't know
12	Over the past 4 weeks, how satisfied did you feel with the quality of your orgasm during sexual stimulation and/or intercourse?	◯ Almost always or always<br>◯ Most of the time<br>◯ Sometimes<br>◯ Never or almost never<br>◯ I'm not sure/I don't know
13	Over the past 4 weeks, have you been able to achieve an orgasm with vaginal penetration?	◯ Almost always or always<br>◯ Most of the time<br>◯ Sometimes<br>◯ Never or almost never<br>◯ I'm not sure/I don't know<br>◯ N/A I have not had vaginal penetration
14	Over the past 4 weeks, have you been able to achieve an orgasm with clitoral stimulation?	◯ Almost always or always<br>◯ Most of the time<br>◯ Sometimes<br>◯ Never or almost never<br>◯ I'm not sure/I don't know
15	Over the past 4 weeks, how satisfied were you with the level of your sexual activity?	◯ Almost always or always<br>◯ Most of the time<br>◯ Sometimes<br>◯ Never or almost never<br>◯ I'm not sure/I don't know

16 Over the past 4 weeks, how satisfied were you with vaginal penetration?

- ◯ Almost always or always
- ◯ Most of the time
- ◯ Sometimes
- ◯ Never or almost never
- ◯ I'm not sure/I don't know
- ◯ N/A I have not had vaginal penetration

17 Over the past 4 weeks, how satisfied were you with the width of your vagina (or the ability of your vagina to accommodate what you are putting in it)?

- ◯ Almost always or always
- ◯ Most of the time
- ◯ Sometimes
- ◯ Never or almost never
- ◯ I'm not sure/I don't know

18 Over the past 4 weeks, how satisfied were you with the depth of your vagina?

- ◯ Almost always or always
- ◯ Most of the time
- ◯ Sometimes
- ◯ Never or almost never
- ◯ I'm not sure/I don't know

19 Over the past 4 weeks, how often did you experience pain during vaginal penetration?

- ◯ Almost always or always
- ◯ Most of the time
- ◯ Sometimes
- ◯ Never or almost never
- ◯ I'm not sure/I don't know
- ◯ N/A I have not had vaginal penetration

20 Over the past 4 weeks, how would you rate your level of pain during vaginal penetration?

- ◯ Severe
- ◯ Moderate
- ◯ Low
- ◯ Very low or absent
- ◯ I'm not sure/I don't know
- ◯ N/A I have not had vaginal penetration

21 Over the past 4 weeks, how often did you experience pain while receiving oral sex?

- ◯ Almost always or always
- ◯ Most of the time
- ◯ Sometimes
- ◯ Never or almost never
- ◯ I'm not sure/I don't know
- ◯ N/A I have not had oral sex

22 Over the past 4 weeks, how often did you experience pain with masturbation?

- ◯ Almost always or always
- ◯ Most of the time
- ◯ Sometimes
- ◯ Never or almost never
- ◯ I'm not sure/I don't know
- ◯ N/A I have not masturbated

23 Over the past 4 weeks, during sexual activity or intercourse, how would you rate the sensitivity of your clitoris?

- ◯ High
- ◯ Moderate
- ◯ Low
- ◯ Very low or absent
- ◯ I'm not sure/I don't know

24 Over the past 4 weeks, during sexual activity or intercourse, how would you rate the sensitivity of your labia majora (outer lips)?

- ◯ High
- ◯ Moderate
- ◯ Low
- ◯ Very low or absent
- ◯ I'm not sure/I don't know

25 Over the past 4 weeks, during sexual activity or intercourse, how would you rate the sensitivity of your labia minora (inner lips)?

- ◯ High
- ◯ Moderate
- ◯ Low
- ◯ Very low or absent
- ◯ I'm not sure/I don't know

26 Over the past 4 weeks, during vaginal penetration, how would you rate the sensitivity of your prostate or erectile tissue within your vagina?

- ◯ High
- ◯ Moderate
- ◯ Low
- ◯ Very low or absent
- ◯ I'm not sure/I don't know

Mental Health Screening - Patient Health Questionnaire - 9

The following 9 questions are a screening for depression. Over the last two weeks, how often have you been bothered by any of the following:

If any of the following questions are triggering please reach out to the study team or contact the number below.
National Suicide Prevention Lifeline at 800-273-8255 and the Crisis Text Line by texting TALK to 741741. Dr. Pope's Office 412-979-2939

		Almost always or always	Most of the time	Sometimes	Never or almost never
1	Little interest or pleasure in doing things	◯	◯	◯	◯
2	Feeling down, depressed, or hopeless	◯	◯	◯	◯
3	Trouble falling or staying asleep, or sleeping too much	◯	◯	◯	◯
4	Feeling tired or having little energy	◯	◯	◯	◯
5	Poor appetite or overeating	◯	◯	◯	◯
6	Feeling bad about yourself - or that you are a failure or have let yourself or your family down	◯	◯	◯	◯
7	Trouble concentrating on things, such as reading the newspaper or watching television	◯	◯	◯	◯
8	Moving or speaking so slowly that other people could have noticed? Or the opposite - being so fidgety or restless that you have been moving around a lot more than usual	◯	◯	◯	◯
9					

Thoughts that you would be better off dead or of hurting yourself in some way	◯	◯	◯	◯

Thank you so much for your time. Again, if any of these questions brought up unpleasant feelings for you, please call the numbers above.

We will reach out to you ASAP to schedule your interview to discuss the survey questions.

THANK YOU!
