# Supplementary material for: Sexual satisfaction and function (SatisFunction) survey post-vaginoplasty for transgender and gender diverse individuals: preliminary development and content validity for future clinical use
Source: Sex Med. 2025 Mar 8;13(1):qfaf011. doi: 10.1093/sexmed/qfaf011 (PMC11890106; doi:10.1093/sexmed/qfaf011)
Supplement: Supplemental_Appendix_qfaf011 [file supplemental_appendix_qfaf011.zip › Supplemental_Appendix_qfaf011/Supplemental Appendix C 32 Question Survey Post Feedback.pdf]

SEXUAL SATISFACTION AND FUNCTION SURVEY

Please fill out all questions below to the best of your ability. You can save and return to the survey using the link in your email if needed. When the survey is completed in its entirety, please click submit at the bottom of the page. Thank you for your time!

Have you been sexually active in the past 4 weeks?<br><br>(select all that apply)	☐ Yes, with a partner(s)<br>☐ Yes, alone<br>☐ No, and I did not want to<br>☐ No, but I wanted to
What type of vaginoplasty did you receive?<br><br>(If you have had more than one, select the one you had most recently)	◯ Zero Depth/Vulvoplasty<br>◯ Shallow Depth<br>◯ Penile Inversion<br>◯ Peritoneal Pull Through<br>◯ Intestinal/Colon<br>◯ Other<br>◯ I'm Not Sure
If Other, Specify	__________________________

Sexual Satisfaction and Function Post-Vaginoplasty

This is a survey that will help assess your sexual satisfaction and function since your vaginoplasty procedure. Each of the following questions will ask you about different aspects of your sexual satisfaction and function and refers to sexual activity.

Sexual Activity = oral sex, penetrative sex, masturbation, sex toys, etc. (alone or with partner(s))
Non-Sexual Activity = dilator use, in office vaginal/pelvic exam, speculum use, etc.

Please answer the following questions based on how you have felt over the past 4 weeks.

1 How comfortable are you with the physical appearance of your external genitalia?

- ◯ Very comfortable
- ◯ Moderately comfortable
- ◯ Slightly comfortable
- ◯ Not comfortable at all
- ◯ I'm not sure/I don't know

2 How comfortable do you or would you feel with your partner(s) seeing your external genitalia?

- ◯ Very comfortable
- ◯ Moderately comfortable
- ◯ Slightly comfortable
- ◯ Not comfortable at all
- ◯ I'm not sure/I don't know
- ◯ I do not have any partner(s)

3 How often does scarring interfere with how comfortable you are with the physical appearance of your external genitalia?

- ◯ Never or almost never
- ◯ Sometimes
- ◯ Most of the time
- ◯ Almost always or always
- ◯ I'm not sure/I don't know

4 Compared to before your procedure, rate your gender dysphoria in relation to your genitalia.

(gender dysphoria means discomfort or stress related to gender)

- ◯ Much lower or absent
- ◯ Lower
- ◯ Higher
- ◯ Much higher
- ◯ I'm not sure/I don't know

5 How often do you feel sexual desire?

(the feeling of wanting a sexual experience, feeling receptive to a partner's sexual initiation, or fantasizing about having sex)

- ◯ Almost always or always
- ◯ Most of the time
- ◯ Sometimes
- ◯ Never or almost never
- ◯ I'm not sure/I don't know

6 How satisfied are you with your level of sexual desire?

- ◯ Very satisfied
- ◯ Moderately satisfied
- ◯ Slightly satisfied
- ◯ Not satisfied at all
- ◯ I'm not sure/I don't know

7 How satisfied are you with the frequency of your sexual arousal?

(the mental/physical feelings of sexual excitement, warmth or tingling in the genitals, muscle contractions)

- ◯ Very satisfied
- ◯ Moderately satisfied
- ◯ Slightly satisfied
- ◯ Not satisfied at all
- ◯ I'm not sure/I don't know

8	How would you rate the intensity of your sexual arousal?	◯ High<br>◯ Moderate<br>◯ Low<br>◯ Very low or absent<br>◯ I'm not sure/I don't know
9	How satisfied are you with the intensity of your sexual arousal?	◯ Very satisfied<br>◯ Moderately satisfied<br>◯ Slightly satisfied<br>◯ Not satisfied at all<br>◯ I'm not sure/I don't know
10	How often do you feel secretions during sexual activity or intercourse without using lubricants?<br><br>(engaging in sexual activity alone or with a partner)	◯ Almost always or always<br>◯ Most of the time<br>◯ Sometimes<br>◯ Never or almost never (I always need to use lubrication)<br>◯ I'm not sure/I don't know<br>◯ N/A I am not sexually active
11	How often do you feel unwanted secretions outside of sexual activity or intercourse?	◯ Never or almost never<br>◯ Sometimes<br>◯ Most of the time<br>◯ Almost always or always<br>◯ I'm not sure/I don't know
12	How often have you been able to have an orgasm when you wanted to?	◯ Almost always or always<br>◯ Most of the time<br>◯ Sometimes<br>◯ Never or almost never<br>◯ I'm not sure/I don't know<br>◯ N/A I am not sexually active
13	How satisfied do you feel with the quality of your orgasm during sexual stimulation and/or intercourse?	◯ Very satisfied<br>◯ Moderately satisfied<br>◯ Slightly satisfied<br>◯ Not satisfied at all<br>◯ I'm not sure/I don't know<br>◯ N/A I am not sexually active
14	How often have you been able to achieve an orgasm with vaginal penetration?	◯ Almost always or always<br>◯ Most of the time<br>◯ Sometimes<br>◯ Never or almost never<br>◯ I'm not sure/I don't know<br>◯ N/A I do not have vaginal penetration
15	How often have you been able to achieve an orgasm with clitoral stimulation?	◯ Almost always or always<br>◯ Most of the time<br>◯ Sometimes<br>◯ Never or almost never<br>◯ I'm not sure/I don't know<br>◯ N/A I do not have clitoral stimulation
16	How often have you been able to achieve an orgasm with anal penetration?	◯ Almost always or always<br>◯ Most of the time<br>◯ Sometimes<br>◯ Never or almost never<br>◯ I'm not sure/I don't know<br>◯ N/A I do not have anal penetration

17	How satisfied are you with the amount of sexual activity you have?	◯ Very satisfied<br>◯ Moderately satisfied<br>◯ Slightly satisfied<br>◯ Not satisfied at all<br>◯ I'm not sure/I don't know<br>◯ N/A I am not sexually active
18	How satisfied are you with the feeling of vaginal penetration?	◯ Very satisfied<br>◯ Moderately satisfied<br>◯ Slightly satisfied<br>◯ Not satisfied at all<br>◯ I'm not sure/I don't know<br>◯ N/A I do not have vaginal penetration
19	How satisfied are you with the width of your vagina?<br><br>(the ability of your vagina to accommodate what you are putting in it)	◯ Very satisfied<br>◯ Moderately satisfied<br>◯ Slightly satisfied<br>◯ Not satisfied at all<br>◯ I'm not sure/I don't know<br>◯ N/A I am not sexually active
20	How satisfied are you with the depth of your vagina?	◯ Very satisfied<br>◯ Moderately satisfied<br>◯ Slightly satisfied<br>◯ Not satisfied at all<br>◯ I'm not sure/I don't know<br>◯ N/A I am not sexually active
21	How often do you experience pain during vaginal penetration?	◯ Never or almost never<br>◯ Sometimes<br>◯ Most of the time<br>◯ Almost always or always<br>◯ I'm not sure/I don't know<br>◯ N/A I do not have vaginal penetration
22	How would you rate the intensity of pain you experience during vaginal penetration?	◯ Very low or absent<br>◯ Low<br>◯ Moderate<br>◯ Severe<br>◯ I'm not sure/I don't know<br>◯ N/A I do not have vaginal penetration
23	How often do you experience pain with clitoral stimulation?	◯ Never or almost never<br>◯ Sometimes<br>◯ Most of the time<br>◯ Almost always or always<br>◯ I'm not sure/I don't know<br>◯ N/A I do not have clitoral stimulation
24	How would you rate the intensity of pain you experience with clitoral stimulation?	◯ Very low or absent<br>◯ Low<br>◯ Moderate<br>◯ High<br>◯ I'm not sure/I don't know<br>◯ N/A I do not have clitoral stimulation

25	How would you rate the sensitivity of your clitoris?<br><br>(sensitivity describes awareness of light touch)	◯ High<br>◯ Moderate<br>◯ Low<br>◯ Very low or absent<br>◯ I'm not sure/I don't know
26	How satisfied are you with the sensitivity of your clitoris?<br><br>(sensitivity describes awareness of light touch)	◯ Very satisfied<br>◯ Moderately satisfied<br>◯ Slightly satisfied<br>◯ Not satisfied at all<br>◯ I'm not sure/I don't know
27	How would you rate the sensitivity of your labia majora (outer lips of the vagina)?<br><br>(sensitivity describes awareness of light touch)	◯ High<br>◯ Moderate<br>◯ Low<br>◯ Very low or absent<br>◯ I'm not sure/I don't know
28	How satisfied are you with the sensitivity of your labia majora (outer lips of vagina)?<br><br>(sensitivity describes awareness of light touch)	◯ Very satisfied<br>◯ Moderately satisfied<br>◯ Slightly satisfied<br>◯ Not satisfied at all<br>◯ I'm not sure/I don't know
29	How would you rate the sensitivity of your labia minora (inner lips of the vagina)?<br><br>(sensitivity describes awareness of light touch)	◯ High<br>◯ Moderate<br>◯ Low<br>◯ Very low or absent<br>◯ I'm not sure/I don't know
30	How satisfied are you with the sensitivity of your labia minora (inner lips of vagina)?<br><br>(sensitivity describes awareness of light touch)	◯ Very satisfied<br>◯ Moderately satisfied<br>◯ Slightly satisfied<br>◯ Not satisfied at all<br>◯ I'm not sure/I don't know
31	During vaginal penetration, how would you rate the sensitivity of your prostate (erectile tissue between bladder and rectum)?<br><br>(sensitivity describes awareness of touch/pressure)	◯ High<br>◯ Moderate<br>◯ Low<br>◯ Very low or absent<br>◯ I'm not sure/I don't know<br>◯ N/A I do not have vaginal penetration
32	How satisfied are you with the the sensitivity of your prostate (erectile tissue between bladder and rectum)?<br><br>(sensitivity describes awareness of touch/pressure)	◯ Very satisfied<br>◯ Moderately satisfied<br>◯ Slightly satisfied<br>◯ Not satisfied at all<br>◯ I'm not sure/I don't know<br>◯ N/A I do not have vaginal penetration
	Any comments or concerns? Please feel free to share any comments about your sexual journey.	__________________________________

Utrecht Gender Dysphoria Scale - Gender Spectrum (UGDS-GS)

For each question, select the response that best describes how much you agree with each statement. Note: Assigned sex means the sex you were assigned at birth and affirmed gender is the gender you currently identify with.

		Disagree completely	Disagree	Neither Agree nor Disagree	Agree	Agree Completely
1	I prefer to behave like my affirmed gender	○	○	○	○	○
2	Every time someone treats me like my assigned sex I feel hurt.	○	○	○	○	○
3	It feels good to live as my affirmed gender.	○	○	○	○	○
4	I always want to be treated like my affirmed gender.	○	○	○	○	○
5	A life in my affirmed gender is more attractive for me than a life in my assigned sex.	○	○	○	○	○
6	I feel unhappy when I have to behave like my assigned sex.	○	○	○	○	○
7	It is uncomfortable to be sexual in my assigned sex.	○	○	○	○	○
8	Puberty felt like a betrayal.	○	○	○	○	○
9	Physical sexual development was stressful.	○	○	○	○	○
10	I wish I have been born as my affirmed gender.	○	○	○	○	○
11	The bodily functions of my assigned sex are distressing for me (erection, menstruation).	○	○	○	○	○
12	My life would be meaningless if I would have to live as my assigned sex.	○	○	○	○	○
13	I feel hopeless if I have to stay in my assigned sex.	○	○	○	○	○
14	I feel unhappy when someone misgenders me.	○	○	○	○	○
15	I feel unhappy because I have the physical characteristics of my assigned sex.	○	○	○	○	○
16	I hate my birth assigned sex.	○	○	○	○	○
17	I feel uncomfortable behaving like my assigned sex.	○	○	○	○	○

18

It would be better not to live, than to live as my assigned sex.	○	○	○	○	○

The Female Sexual Distress Scale-Revised (FSDS-R; revised 2005): Screening Questionnaire for Measuring Sexually Related Personal Distress in Women With Female Sexual Dysfunction (FSD)

Below is a list of feelings and problems that women sometimes have concerning their sexuality. Please read each item carefully, and circle the number that best describes how often that problem has bothered you or caused you distress during the past 30 days including today.

		Never	Rarely	Occasionally	Frequently	Always
1	Distressed about your sex life	○	○	○	○	○
2	Unhappy about your sexual relationship	○	○	○	○	○
3	Guilty about sexual difficulties	○	○	○	○	○
4	Frustrated by your sexual problems	○	○	○	○	○
5	Stressed about sex	○	○	○	○	○
6	Inferior because of sexual problems	○	○	○	○	○
7	Worried about sex	○	○	○	○	○
8	Sexually inadequate	○	○	○	○	○
9	Regrets about your sexuality	○	○	○	○	○
10	Embarrassed about sexual problems	○	○	○	○	○
11	Dissatisfied with your sex life	○	○	○	○	○
12	Angry about your sex life	○	○	○	○	○
13	Bothered by low sexual desire	○	○	○	○	○

If any of these questions have triggered distress for you, please feel free to reach out to a psychologist who specializes in gender dysphoria. At UH, you can schedule with Dr. R. Brian Denton by calling (216) 844-3009.

Other resources: National Suicide Prevention Lifeline at 800-273-8255 and the Crisis Text Line by texting TALK to 741741.

THANK YOU!
